# NgAgo: a hope or a hype?

**DOI:** 10.1007/s13238-016-0344-8

**Published:** 2016-11-17

**Authors:** Xiaoxue Zhang

**Affiliations:** Protein & Cell Editorial Office, Beijing, 100101 China

For decades of biomedical research, scientists have been enchanted by, and relentlessly hunted for, powers of modifying the inheritable genomic information in particular cell types or in living organisms. Such technological capabilities, generally known as gene- or genome-editing, hold great promises for both scientific research and medicine. Indeed, the discovery of restriction enzymes in 1960s has led to the birth of recombinant DNA technique and the bloom of modern biotechnology, and the development of TALEN and CRISPR systems in the past few years have launched the new era of genome-editing research and raised the hope for therapeutic applications.

Despite such exciting achievements we have witnessed, enormous challenges still remain for further advances of genome-editing techniques, in particular, to enhance the editing efficiency and to eliminate the off-target events. In May 2016, Gao et al. reported that an archaebacterium-derived DNA nuclease named NgAgo could precisely recognize and efficiently edit the genomic DNA of mammalian cells in a DNA oligonucleotide-guided manner. Given the researchers’ desire for new genome-editing tools, it is not unexpected that this report has drawn immediate attentions and generated broad enthusiasms in the scientific community. For instance, it was suggested that NgAgo could perform as an important, or an even superior alternative to the CRISPR system whose editing process is guided by a short sequence of RNA instead of DNA.

However, as a bitter twist of the initial excitements, concerns of the reproducibility of NgAgo genome-editing technique have been accumulating in the past few months. Criticisms that NgAgo did not work in genome-editing experiments as originally reported can be found on many online scientific forums. Also, researchers have begun to discuss, openly or in private, their failures to reproduce the key findings of Gao et al. With this ongoing controversy of NgAgo genome-editing technique, *Protein & Cell* has decided to publish in this issue a scientific letter, jointly composed by 20 independent research groups from different institutions worldwide, that refutes the initial report by Gao et al. In this letter, the authors show that NgAgo exhibits little, if any, editing activities towards the genomic DNA of mammalian cells under a range of experimental conditions. *Protein & Cell* notes that, although this controversy of NgAgo technique has been fermenting for months, most of criticisms were not presented in formal and scholarly manners, particularly, in a peer-reviewed scientific journal, which has significantly limited the research community from assessing the dueling claims from the two sides. The editors, therefore, believe and wish that publication of this collection of contradicting results could set off the momentum in the research community to scrutinize the potentials of NgAgo, and to venture forwards to further expanding the toolbox of genome-editing technique.

